# My journey after a mild infection with COVID-19: I want my old brain back

**DOI:** 10.1590/0004-282X-ANP-2022-0062

**Published:** 2022-08-08

**Authors:** Clarissa Lin Yasuda

**Affiliations:** 1 Universidade Estadual de Campinas, Departamento de Neurologia e Neurocirurgia, Campinas SP, Brazil.; 2 Fundação de Amparo à Pesquisa do Estado de São Paulo, Brazilian Institute of Neuroscience and Neurotechnology, São Paulo SP, Brazil.

**Keywords:** COVID-19, Cognitive Dysfunction, Post-COVID Syndrome, Fatigue, COVID-19, Disfunção Cognitiva, Síndrome Pós-COVID, Fadiga

## Abstract

Although neurocognitive dysfunction has been observed after infection by SARS-CoV-2, few studies have detailed these alterations or demonstrated their impact on daily life activities and work. Here, I describe the sequence of events following a mild COVID-19 infection in August 2020 (which now is described as “post-COVID syndrome”) and comment on my ensuing limitations associated with cognitive difficulties, headache, fatigue and sleepiness. Furthermore, I discuss the efforts that I have made to recover from my infection since its beginning and the strategies adopted for living with persistent restrictions in terms of cognitive performance.

## RESUMO

Embora a disfunção neurocognitiva tenha sido observada após a infecção por SARS-Cov2, poucos estudos detalharam essas alterações ou demonstraram seu impacto nas atividades de vida diária e trabalho. Aqui eu descrevo a sequência de eventos após uma infecção leve por COVID-19 em agosto de 2020 (agora considerada Síndrome pós-COVID) e comento as limitações associadas às dificuldades cognitivas, dor de cabeça, fadiga e sonolência. Além disso, eu caracterizo o esforço de recuperação desde o início e as estratégias para conviver com restrições persistentes em termos de desempenho cognitivo.

I am a 46-year-old neurologist working as an assistant professor of neurology at a tertiary hospital in Brazil. In March 2020, I designed the NeuroCovid study at the University of Campinas, combining MRI analyses, neuropsychological and clinical evaluation and immunological analyses. I chose this multimodal approach because I was involved in epilepsy research and neuroimaging (neuroimmunology of multiple sclerosis was the focus of my undergraduate research project for five years) before the pandemic. We started evaluating post-COVID volunteers in July 2020, and I became infected in August 2020. It was a mild infection without fever, dysgeusia, anosmia or respiratory symptoms. I presented headache (worsened migraine), severe abdominal pain, sleepiness, diarrhea, vomiting and hiccups; it was winter, and I felt unusually cold in Campinas, Brazil (I have experienced -45 Celsius during winter in Canada). These symptoms terminated within ten days (except the headache), and I truly believed it was over. I was happy to be back at the hospital to continue to evaluate the post-COVID subjects. 

Approximately 3-4 weeks after the acute stage, I knew my migraine had worsened, and I could not tolerate the atenolol (50 mg) that I had been using as a prophylactic for migraine and hypertension treatment. This intolerance was mainly during swimming practice. At the same time, I noticed excessive somnolence during the day, associated with an increase of 1.5-2.0 hours of night sleep. It felt awkward because I had always been an energetic person who usually had 6-7 hours of sleep. Now I needed 8-9 hours of rest, yet I was also experiencing daytime somnolence. I realized that I was having difficulties writing a grant proposal and performing more complex statistical analyses. I tried modafinil because I had daytime somnolence; it improved the somnolence but not my cognitive performance. 

At that moment, I realized that I had some cognitive dysfunction impairing my academic tasks. There were no difficulties with daily tasks or patient care, except the fatigue and somnolence. I knew my difficulties were mainly in relation to more complex tasks (sophisticated statistical models and writing papers). I missed deadlines; I could not write papers as fast as before. I also missed some meetings (I was distracted and sleepy) and could no longer perform simultaneous tasks, as I used to. I noticed that I had slower brain processing speed and inattention. I had to struggle with excessive daytime sleepiness and fatigue, along with a combination of difficulties and frustration associated with symptoms of depression and anxiety.

However, I am grateful for the support of my team of postgraduate students and colleagues. I never tried to hide my symptoms and difficulties; they have supported me since the beginning. We have discussed the drugs that I have been trying because this is a new virus^1^ and almost nothing about any persistent neurocognitive dysfunction that it might cause is known^2^. I decided to use levetiracetam for migraine prophylaxis^3^ because I knew it could somehow improve my brain connectivity^4^. It was a good choice because it reduced the migraine attacks and somehow reduced the excessive night sleep by 0.5-1.0 hour. Nevertheless, I had to adjust the dose due to worsening symptoms of depression with the high dose; therefore, I combined it with an antidepressant (agomelatine).

At the end of 2020, I realized I had to adjust to the new situation and slow down my pace of life and academic activities. I had already resumed my swimming workouts (2000 m, with variable intensity and snorkel) 2-3 times/week, combined with Pilates and one day of strength exercise. There was hope that intensive aerobic exercise would help recover the cognition^5^.

In January 2021, I decided to try low doses of lisdexamfetamine, which finally helped me work, although not as fast or efficiently as before COVID-19. However, I do not tolerate it well and only use it 2-3 times on weekdays, especially on busy days at work. I manage my routine with an online agenda (which I never needed before). I need to sleep more than before; I do my swimming workouts and respect my fatigue and tiredness. I take drugs that I never imagined I would need. After each dose of vaccine (COVID-19 and meningitis), it was intriguing that I felt I lost each slight improvement for approximately four weeks. 

After 16 months (end of 2021), I finally thought that part of my old brain was coming back. Some automatic connections (part of my personality) that I had lost are now back, such as the names of several patients and their histories. It seems that I have a slight improvement in brain processing speed. Roughly, if I lost 30% of my natural speed after COVID-19, I think that I have recovered 10%. It is sad and disappointing. It is embarrassing and painful not to recognize myself.

I believe that transparency about all the uncertainties surrounding the new coronavirus is needed^1^. Not much data on the negative impact of prolonged symptoms is available, nor are the effects of SARS-CoV-2 on either the central or the peripheral nervous system understood^6^. I am frightened because previous studies showed the vulnerability of the hippocampus to coronavirus^2^. I have studied hippocampal atrophy in epilepsy for almost 20 years, and now I face the risk that the new coronavirus will act as an initial precipitating injury and eventually cause hippocampal atrophy and epilepsy. Or it may accelerate a neurodegenerative process with dementia^2^. 

In an article now submitted (in collaboration with other researchers), we already observed grey matter atrophy in the orbitofrontal cortex of mildly infected individuals (81 individuals with an average age of 37 years), which was associated with poor performance in the trail-making test^7^. This has also been recently reported in a larger group (401 subjects with an average of 62 years)^8^. In our study^7^, we identified that there was higher frequency of symptoms of anxiety and depression approximately two months after the COVID-19 diagnosis, which was in line with findings from an extensive study of survivors^9^. 

I fear for the numerous survivors of COVID-19 who do not have access to medical attention for their post-COVID symptoms. I have received several emails from individuals with similar (or worse) symptoms; they complain that most physicians do not understand or believe in the multitude of symptoms. It is frustrating because I know all these symptoms are real and compromise our life and work. As well described previously by other doctors^1^ and in another study^10^, the mental health system needs to become prepared to receive survivors with different neuropsychiatric symptoms, including anxiety and depression. 

Longitudinal studies on these symptoms need to be continued in order to more precisely understand the predisposing factors for neurocognitive and cerebral alterations associated with SARS-CoV-2. Given the uncertainties about the underlying mechanisms, COVID-19 cannot be prevented or treated specifically; nor can it be predicted whether the alterations that it causes are temporary, permanent or progressive. 

We are starting a new project to offer cognitive rehabilitation to survivors with post-COVID dysfunction. It is a small, local project. Nevertheless, we expect to bring hope to individuals and simultaneously collect multimodal data to understand possible mechanisms behind neurocognitive dysfunction.
